# Tropical Diseases in the Navy

**Published:** 1921-02-26

**Authors:** 


### Tropical Diseases in the Navy.

A variety of interesting and not generally realised facts were brought to notice by Surgeon Rear-Admiral Bassett-Smith in his recent lectures at the Middlesex Hospital. The geographical conditions favourable to malaria are fairly perfectly known, but temperature and humidity of the atmosphere are in reality the first consideration. In dry air and low temperatures the disease is unknown. For example, there is no malaria in or on the borders of the Sahara, in spite of its position between the parallels of latitude limiting the natural zone of the disease. So local is the climatic effect that, in the Ægean during the war, naval ratings suffered from malaria in more or less direct proportion to their proximity to the coast-line. Lighters tied up to the shore often had every man down, whereas, a point in practical prevention, those anchored even half a mile off-shore had not a single case, since the prevalent evening breeze was from the sea, landwards. As far as the Navy is concerned, the highest proportion of cases occurred, as might therefore be expected, in coastal air stations, and the return of some of these trained men to the corresponding units in Great Britain undoubtedly accounts for the excessive number of malaria cases in these localities.

An important misconception, which these lectures should remove, concerns Malta or Mediterranean fever. The lecturer claims that it should always be termed undulant fever, because, although it is specially prevalent in Malta, it does not confine itself either to this place or to littoral areas, being well established inland in India, the Punjab, the Yang-Tse Valley, the Philippines, Northern Nigeria, and Uganda. After the Boer war the disease became common in South Africa and the Transvaal, where cattle were relatively few and quantities of goat's milk used.

The long incubation period of this disease is curious in its effect, in that men passing through these areas are often infected, the onset occurring in other stations, and often a naval rating will apparently run through the whole of his time in the Mediterranean without obvious disease, only to develop it on returning to England.

It is not only the Maltese goat which carries the bacteria, although at home or exported he is the worst offender. Any goats are susceptible, and from these it may spread to cows, mules, horses, and even dogs. Recently infection has frequently been traced to fresh native-made cheese, and the lecturer met the case of a Greek officer, who suffered from a most severe attack, where the source of infection was undoubtedly ice-cream taken in Algeria.

As far as the Services go, however, compulsory sterilisation of all milk has almost abolished the disease, and in this way an enormous saving of money, time, and distress has been brought about. In 1904 there were 430 cases in the Navy, representing 28,454 days' sickness and nine deaths. In 1907 the figure was reduced to twelve for the fleet, but at that time a high rate remained among the civil population. During the Great War there were in all only four naval cases known to occur. Would that all infectious disease was thus easily and efficiently controlled!

The third lecture was devoted to sleeping sickness, and the chief value from a public point of view lay in the definite exposition of the fallacy that there is any connection between the "sleeping sickness" lately apparent in England and that of Tropical Africa. The causal organism of the latter is well known, and it is probable that the natives have been subject to it for a long period, and have acquired some degree of immunity. This is still a doubtful point however, but it is remarkable how many animals can act as the hosts of the trypanosomes, as the organisms are called, and yet suffer no ill-effects. The disease is absolutely and entirely different from encephalitis lethargica, which is apparently due to an ultra-microscopic "filter-passer."

